# Teens Using Screens for Help: Impact of Suicidal Ideation, Anxiety, and Depression Levels on Youth Preferences for Telemental Health Resources

**DOI:** 10.2196/13230

**Published:** 2019-06-21

**Authors:** Tammy Toscos, Amanda Coupe, Mindy Flanagan, Michelle Drouin, Maria Carpenter, Lauren Reining, Amelia Roebuck, Michael J Mirro

**Affiliations:** 1 Parkview Research Center Parkview Health Fort Wayne, IN United States; 2 Department of Psychology Purdue University Fort Wayne, IN United States

**Keywords:** adolescent, students, telemedicine, mental health, suicidal ideation, depression, anxiety, health resources, online social networking, mental health services, help-seeking behavior

## Abstract

**Background:**

High rates of mental illness, stress, and suicidality among teens constitute a major public health concern in the United States. However, treatment rates remain low, partially because of barriers that could be mitigated with tech-based telemental health (TMH) resources, separate from or in addition to traditional care.

**Objective:**

This study aimed to analyze TMH resource usage by high school students to establish current user characteristics and provide a framework for future development.

**Methods:**

A total of 2789 students were surveyed regarding demographics, recent anxiety and depression symptoms, suicidality, and stress; people with whom they could openly and honestly discuss stress or problems, and prior TMH use. Logistic regression models and a general linear model were used to test relationships between variables.

**Results:**

Overall, 30.58% (853/2789) and 22.91% (639/2789) of students reported moderate to severe anxiety and depression symptoms, respectively, in the past 2 weeks; 16.24% (414/2550) had seriously considered suicide in the past year, consistent with national averages. Meanwhile, 16.03% (447/2789) of students had previously used at least 1 of 4 types of TMH resources (ie, self-help, anonymous chat, online counselor, or crisis text line). Teens reporting depression symptoms, higher stress, or suicidality were less likely to talk to a parent about stress or problems and more likely to tell no one. Suicidality was related to the use of all 4 types of TMH resources. Depression symptoms were related to the use of anonymous chat and crisis text line, and those with higher stress were more likely to have used an online counselor. Those reporting anxiety symptoms were less likely to have no one to talk to and more likely to have used a self-help resource.

**Conclusions:**

Youth struggling with mental health symptoms, some of whom lack real-life confidants, are using existing TMH support, with resource preferences related to symptoms. Future research should consider these preferences and assist in the creation of specialized, evidence-based TMH resources.

## Introduction

### Background

With rates rising over the last decade [1], suicide is the second leading cause of death among adolescents and young adults aged 15 to 24 years [2]. In 2016, 17.2% of high school students reported serious suicidal thoughts, 13.6% made a plan, and 7.4% attempted suicide. The rate of suicidal ideation among adolescents with depression is much higher, with some estimates as high as 40 to 50% [3,4]. Depression itself is a public health concern for youth, as well: in 2016, 12.8% of adolescents aged 12 to 17 years reported experiencing a major depressive episode in the last year [5]. Another survey from the same year suggested an even higher prevalence of subclinical depression symptoms, with 31.5% of high school students feeling sad and hopeless nearly every day in the past 2 weeks [6]. Anxiety disorders are also present among teens aged 13 to 18 years at high rates (31% lifetime prevalence) [7], and, like suicidality and depression, affect females more than males [5-7].

As with all health care, there are barriers to mental health service access. Less than 30% of teens experiencing suicidality and 40% of teens experiencing major depression seek professional treatment [5,8], and only about a third of teenagers with *any* mental health issues are treated by a specialist [9]. Treatment rates are even lower among minorities [3,8] and those with geographical constraints [4,8]. In addition to physician shortage [10-12] and patient financial struggles [8,10], stigma is a major factor discouraging teens from seeking mental health treatment, which includes reticence with sharing symptoms with a parent to initiate the process [13-17]. This may disproportionately affect male teens, who are less likely to seek help for suicidal ideation than females [8,15]. Teens also may lack awareness of their symptoms or treatment options [13,16-18], perceive high stress as normal [8,13], have confidentiality concerns [8,13-16], or feel that adults will not understand their problems [13,15].

Youth may instead seek out informal sources of help, such as friends or the internet [13-15,19,20], or attempt to handle their problems alone [8,13-15,20,21]. Those endorsing self-reliance are more likely to be depressed and suicidal and less likely to seek traditional care; however, they are more likely to seek support from anonymous Web-based sources such as forums, chat rooms, or support groups [20]. Some adolescents and young adults report that social interaction on the internet enhances their real-life friendships and contributes to a feeling of social connectedness [22-24]; however, social media can also be a source of stress or negative feelings, and problematic use (ie, that which mirrors behavioral addiction) is associated with depression and anxiety [21,25,26]. Those with higher levels of depression and anxiety are also likely to share their stresses on social media [21,27], as this venue may provide greater control over social interaction with nonjudgmental peers [28]. With regard to social media as a conduit to formal mental health care, 1 study showed that almost 63% of youth were open to a provider proactively contacting them via social media, and 70% expressed a favorable view of receiving professional advice on the internet [27]. However, some view social media as personal space where they do not want provider involvement, citing stigma and confidentiality as concerns [29].

Researchers have suggested that digital health interventions could be particularly successful among younger individuals because of their frequent technology usage [30]. Indeed, 95% of American teens own smartphones and nearly half use the internet “almost constantly,” [22] with those aged 13 to 18 years spending an average of over 6.5 hours per day with screen media, including over 2.5 hours of social media, video-chatting, browsing the Web, or engaging in other computer or mobile activities besides gaming or watching video [31]. Although the health care industry has been slower to utilize the full potential of the internet compared with other sectors [32], many digital health innovations (ie, electronic health, mobile health, and telehealth) have been developed over the last decade to supplement or deliver care [30]. Such innovations have been used successfully with youth to improve chronic disease management [33-36], assist with symptom and behavior monitoring [37,38], supplement face-to-face treatment [39,40], and provide health education [41,42]. In recent years, Web-based services specifically designed to support mental health (ie, telemental health, or TMH) have been developed as well [43]; however, thus far, these resources have not been integrated within the health system, which represents a significant opportunity for improved care [44]. TMH refers to a range of services provided or accessible via communications technologies, such as videoconferencing administered by professionals [11], platforms to mutually discuss problems and seek peer support (eg, chat rooms), and self-help mobile apps [43]. These resources are numerous—recent estimates show over 10,000 commercially available mental health apps, for example—but are not always evidence-based or formally evaluated [45].

At present, data regarding effectiveness and perceptions of TMH resources are both mixed and somewhat limited [17,30,39,46-51]. Upon reviewing trials of TMH resources, researchers note methodological difficulties that inhibit definitive conclusions about efficacy, cost-effectiveness, and clinical applicability [30,52]. However, extant literature suggests that TMH resources based in cognitive behavioral therapy principles may improve symptoms in teens with mild to moderate depression and anxiety [30,48] and that self-monitoring mobile apps, particularly in addition to traditional treatment, may benefit individuals struggling with stress, anxiety, and depression [39,52-55]. In one qualitative study, users discussed the contrasting benefits of both face-to-face and Web-based support (ie, the website 7 Cups of Tea): face-to-face support is administered by a trained professional with whom a patient has a relationship, whereas the website allows anonymous real-time support from people who sympathize with the users’ problems [49]. Researches also note ongoing concerns about the true potential of these resources to expand access to mental health care, noting that some studies have found a lack of user interest, even among younger age groups, and that not all providers endorse this type of nontraditional care [30]. However, many individuals lack knowledge of these resources, and opinions may become more positive with increased information and awareness [46,47,56].

### Objective

Due to the novelty and ever-evolving nature of communication technology, ongoing research of TMH resources is necessary to direct continual development [30,43,48,57-60]. In this study, we investigated the relationships between depression, suicidal ideation, stress, anxiety, communication preferences, and TMH use, with the intent to establish use patterns, especially among at-risk youth, and provide a framework for development and implementation of future technologies.

## Methods

### Sample and Data Collection

Participants were from 4 northwestern Indiana high schools (2 suburban, 2 rural). These schools were representative of the general area of study, which contains 1 midsize city with much suburban sprawl, surrounded by several large rural counties. Surveys were conducted during educational assemblies at each school in February and March 2017. Schools provided detailed study information to parents at least 2 weeks before each event. Parents either passively consented or opted out on their child’s behalf; students also completed an age-appropriate consent or assent process directly before the survey.

**Figure 1:**
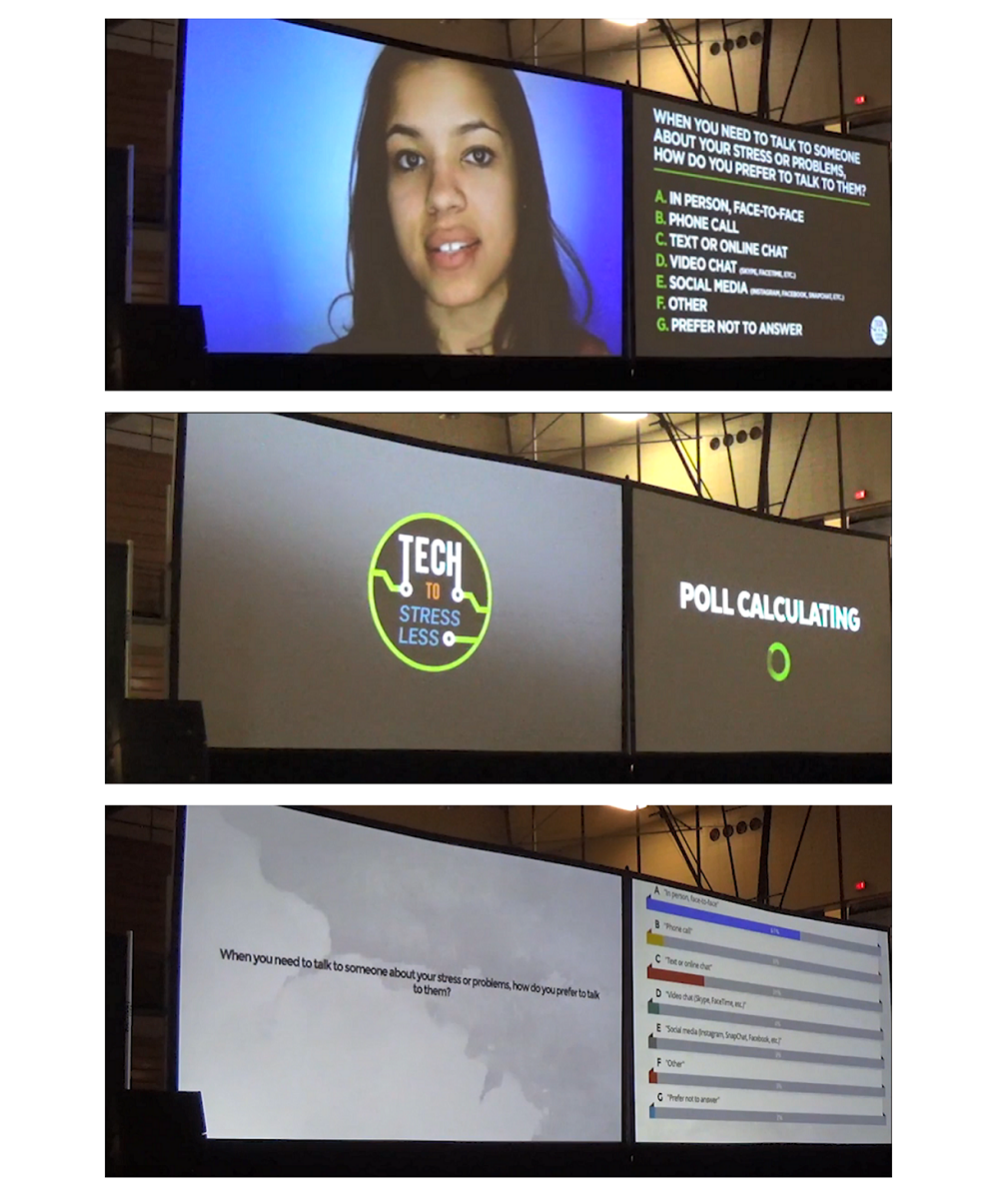
Example screen images from the live survey events.

Students assembled into their school’s auditorium or gymnasium and connected their tablet, laptop, or mobile phone to the secure local Wi-Fi network provided by the study team. Survey questions were presented via prerecorded video, integrated within an hour-long media-rich educational presentation. In addition to the large screen displayed to the group, questions were shown on students’ devices, on which they responded confidentially. Anonymous aggregate responses were displayed to the group after all answers for each question had been recorded, contributing to a larger message of stigma reduction within the presentation. See Figure 1 for screen images.

A more detailed description of these events is available in our methodology paper regarding the use of immediate response technologies to gather health data from youth [61]. The events were engineered by a contracted company specializing in audiovisual presentations, which was vetted by the research institution’s legal department. All procedures were approved by the research institution’s institutional review board (PRC15-1001).

The original sample included 3412 high school students. We removed responses from 168 students who only completed the practice questions, 434 students who stopped the survey before the 30th question, and 27 students who responded “prefer not to answer” or did not respond for 80% or more of the questions, leaving a final sample of 2789. To maintain representativeness in the sample, participants who provided partial data were retained where possible (n=1667), and analysis was performed with pairwise deletions, resulting in varying sample sizes across the results.

### Measures

The 35-question survey began with demographic questions, including age, race, and gender and included the measures below. Due to the sensitive nature of some measures, participants could select “prefer not to answer” or skip any question, with the exception of an initial question regarding age (ie, minor status) to determine consent versus assent.

*Depression* and *anxiety* were measured with the Patient Health Questionnaire-4 (PHQ-4) [62], a validated 4-item measure of depression and anxiety [63,64] in young adults [65], which includes the 2 items in the PHQ-2 plus 2 items from the Generalized Anxiety Disorder 7-item (GAD-7) scale, both of which have been validated as appropriately sensitive and specific measures of detecting depression and anxiety in adolescents aged 13 to 17 years [66,67]. Students used a 4-point Likert scale (0=*not at all*, 3=*nearly every day*) to indicate how often they had experienced anxiety (items 1 and 2) and depression (items 3 and 4) symptoms in the last 2 weeks. These items were summed to create a total PHQ score, as well as depression and anxiety subscale scores, in which a score of 0 to 2 indicates no or mild symptoms, and a score of 3 to 6 indicates moderate or severe symptoms.

*Suicidality* was assessed with 1 item from the Youth Risk Behavior Surveillance System survey [68], a validated measure of recent suicidal thoughts and behaviors in adolescents [69]. Participants were asked, “In the last 12 months, did you ever seriously consider attempting suicide?” and responded *yes* (1) or *no* (0).

*Stress level* was measured with 1 item, adapted from the American Psychological Association’s Stress in America survey [70], that asked students to rate their stress level over the past month on a scale of 0 to 10, where 0= *no stress* and 10= *a great deal of stress.*

*Communication preferences* were established by asking students *with whom they could openly and honestly discuss stress or problems* with multiple response options (of which they could select any or all), including *parent or guardian*; *friend*; *teacher, guidance counselor, or school staff*; *health professional; other adult; someone else;* or *no one*.

*Prior use of TMH resources* was measured with 4 questions regarding use of *anonymous online chat, self-help resources, online therapist or counselor,* and *crisis text line*. To inform students of available resources and improve the sensitivity of these questions, the presentation included audiovisual educational information about each type, including specific websites, apps, and services. Students indicated prior use with “yes, and it was helpful,” “yes, but it was not helpful,” “maybe, I’m not sure” or “no.” Both “yes” answers were combined for analyses predicting prior use.

### Statistical Analysis

Summary statistics were calculated for demographics, suicidality, PHQ-4 anxiety and depression scores, stress, and previous TMH use. To explore bivariate relationships between predictors and outcomes, zero-order correlations were computed (tetrachoric and polychoric for categorical variables, and Pearson coefficients for continuous variables). A total of 3 series of statistical models were tested. First, to understand how covariates were related to mental health outcomes, demographic characteristics (age, gender, race) were entered into separate logistic regression models predicting suicidality, moderate or severe anxiety, and moderate or severe depression; for stress, the same set of covariates were tested using a general linear model. Second, demographics and mental health outcomes were related to individuals with whom students felt they could openly and honestly discuss problems—parents, friends, or no one—using separate logistic regression models. Third, separate ordinal logistic models were used to relate demographics, suicidal ideation, anxiety, depression, and stress to students' previous use of the aforementioned 4 types of TMH resources (3= *yes*, 2= *maybe*, 1= *no*). Analyses were conducted using SAS software 9.4. (SAS Institute Inc). SAS and all other SAS Institute Inc product or service names are registered trademarks or trademarks of SAS Institute Inc, Cary, NC, United States.

## Results

### Demographics and Mental Health

**Figure 2:**
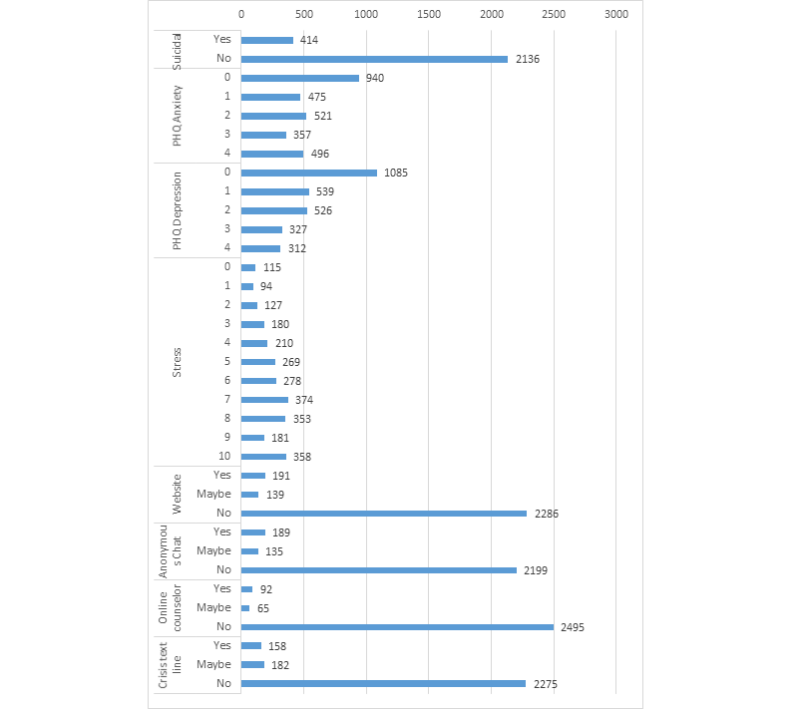
Histogram of number of responses or scores for suicidality, Patient Health Questionnaire (PHQ) anxiety, PHQ depression, stress, and website, anonymous online chat, online counselor, and crisis text line use for telemental health.

As shown in Table 1, the sample was 51.70% (1442/2789) female and 62.60% (1746/2789) white with an average age of 16.09 years (SD 1.20; range=13-19). In addition, 16.24% (414/2550) reported seriously considering a suicide attempt in the previous 12 months, and 30.58% (853/2789) and 22.91% (639/2789) of the sample reported moderate to severe anxiety (mean 1.64, SD 1.49; range=0-4) and depression symptoms (mean 1.37, SD 1.38; range=0-4), respectively, in the last 2 weeks. On average, students reported 6.06 on the stress scale (SD 2.83; range=0-10). Figure 2 includes a histogram for suicidality responses, scores for the PHQ anxiety and depression scales, stress levels, and previous TMH use.

In preliminary analyses examining zero-order correlations for demographics with mental health outcomes, all correlations were low (*r*<.30). Across these mental health outcomes, gender had highest correlations with suicidality (*r*=–.10), anxiety (*r*=–.30), depression (*r*=–.15), and stress (*r*=–.30). Consistently, as shown in Table 2, females compared with males (*P*<.001) and other gender compared with females (*P*<.001) were significantly more likely to report symptoms of anxiety and depression and considering suicide. Similarly, females reported more stress than males (*P*<.001). White students were more likely to report experiencing anxiety (*P*<.001) and stress (*P*=.01) than minorities. Age was also positively related to anxiety (*P*=.003) and stress (*P*=.04).

**Table 1:** High school sample characteristics.

Characteristic		n (%)
**Age (years)**		
	13-14	235 (8.43)
	15-16	1514 (54.30)
	17-19	1039 (37.27)
**Race**		
	White	1746 (62.60)
	Black	342 (12.26)
	Hispanic American or Latino	211 (7.57)
	Other	339 (12.15)
	Prefer not to answer	151 (5.41)
**Gender**		
	Male	1266 (45.39)
	Female	1442 (51.70)
	Other	81 (2.90)
Seriously considered suicide in the last 12 months		414 (16.24)
Moderate or severe anxiety symptoms last 2 weeks		853 (30.58)
Moderate or severe depression symptoms last 2 weeks		639 (22.91)
**Stress (0-10)**		
	0-3	516 (20.32)
	4-7	1131 (44.55)
	8-10	892 (35.13)

**Table 2:** Binary logistic regression analyses for combined demographics predicting suicidality, anxiety, and depression and generalized linear model analysis for combined demographics predicting stress.

Predictor		Seriously considered suicide in the last 12 months (n=2427)		Moderate or severe anxiety in the last 2 weeks (n=2637)		Moderate or severe depression in the last 2 weeks (n=2637)		Average stress during past month (n=2403)	
		B^a^ (SE^b^)	OR^c^ (95% CI^d^)	B (SE)	OR (95% CI)	B (SE)	OR (95% CI)	B (SE)	Wald 95% confidence limit
Intercept		–0.80 (0.76)	—^e^	–1.94^f^ (0.60)	—	–1.87^f^ (0.63)	—	5.50^f^ (0.73)	4.06, 6.94
Age		–0.04 (0.05)	0.96 (0.88-1.06)	0.11^f^ (0.04)	1.12 (1.04-1.20)	0.06 (0.04)	1.06 (0.98-1.14)	0.09^f^ (0.05)	0.005, 0.18
**Race**	
	Not white	–0.06 (0.12)	0.94 (0.74-1,19)	–0.48^f^ (0.10)	0.62 (0.51-0.75)	0.14 (0.10)	1.15 (0.94-1.39)	–0.29^f^ (0.11)	–0.52, –0.07
**Gender**	
	Male versus female	–0.77^f^ (0.12)	0.46 (0.37-0.59)	–1.34^f^ (0.10)	0.26 (0.22-0.32)	–0.84^f^ (0.10)	0.43 (0.36-0.53)	–1.88^f^ (0.11)	–2.10, –1.67
	Other versus female	1.54^f^ (0.29)	4.68 (2.65-8.28)	1.08^f^ (0.29)	2.94 (1.68-5.14)	1.03^f^ (0.27)	2.81 (1.65-4.78)	0.91^f^ (0.39)	0.14, 1.68

^a^Unstandardized parameter estimate.

^b^SE: standard error.

^c^OR: odds ratio.

^d^CI: Confidence Interval.

^e^Not available.

^f^Represents significant findings, *P*<.05.

### Communication Preferences

More students indicated that they could openly and honestly discuss stress or problems with friends (1874/2682, 69.87%) than with parents or guardians (1204/2682, 44.89%); teachers, guidance counselors, or school staff (360/2682, 13.42%); other adults (270/2682, 10.07%); health professionals (204/2682, 7.60%); or someone else not listed (281/2682, 10.48%). Unfortunately, 19.35% (519/2682) of students reported that they could talk with no one about their stress or problems. The largest zero-order correlation coefficients were observed between talking with a parent and previous suicidality (*r*=–.38), anxiety (*r*=–.26), depression (*r*=–.33), and stress (*r*=–.27). Consistently, as shown in Table 3, students who were not white (*P*=.03), male (*P*=.001) with previous suicidality (*P*<.001) and more depression symptoms (*P*<.001) and stress (*P*=.01) were less likely to report that they could talk open and honestly with parents. All other correlations were less than .25. Students who were not white (*P*<.001), male (*P*<.001) with depression symptoms (*P*=.04) were less likely to talk with friends in an open and honest fashion. Finally, students who were not white (*P*=.02), male (*P*<.001), other gender (*P*<.001) with depression symptoms (*P*<.001) and higher stress levels (*P*<.05) were more likely to report that they could talk to no one; interestingly, students with more anxiety were significantly less likely to report talking to no one (*P*=.047).

**Table 3:** Binary logistic regression analyses for combined demographics, suicidality, depression, anxiety, and stress predicting talking with friends, parents, or no one (n=2168).

Predictor		Talk to friend		Talk to parent or guardian		Talk to no one	
		B^a^ (SE^b^)	OR^c^ (95% CI^d^)	B (SE)	OR (95% CI)	B (SE)	OR (95% CI)
Intercept		0.96 (0.67)	—^e^	0.90 (0.62)	—	–2.07^f^ (0.79)	—
Age		0.02 (0.04)	1.02 (0.94-1.11)	–0.001 (0.04)	1.0 (0.93-1.08)	–0.03 (0.05)	0.98 (0.89-1.07)
Race: not white		–0.64^f^ (0.10)	0.53 (0.43-0.64)	–0.22^f^ (0.10)	0.81 (0.67-0.98)	0.28^f^ (0.12)	1.32 (1.05-1.68)
**Gender**	
	Male versus female	–0.37^f^ (0.11)	0.69 (0.56-0.85)	–0.32^f^ (0.10)	0.73 (0.60-0.88)	0.49^f^ (0.13)	1.63 (1.28-2.09)
	Other versus female	–0.55 (0.34)	0.58 (0.30-1.12)	–0.83^g^ (0.42)	0.44 (0.19-1.00)	1.31^f^ (0.34)	3.70 (1.89-7.22)
Suicide: Yes		–0.13 (0.15)	1.14 (0.85-1.53)	–.59^f^ (0.15)	1.80 (1.34-2.41)	0.31^g^ (0.16)	1.37 (1.00-1.87)
Depression		–0.10^f^ (0.05)	0.90 (0.82-0.99)	–0.24^f^ (0.05)	0.79 (0.72-0.86)	0.28^f^ (0.06)	1.32 (1.18-1.48)
Anxiety		0.005 (0.05)	1.01 (0.91-1.11)	–0.06 (0.04)	0.95 (0.87-1.03)	–0.13^f^ (0.06)	0.88 (0.79- 0.99)
Stress		0.04^g^ (0.02)	1.05 (1.00-1.09)	–0.05^f^ (0.02)	0.95 (0.91-0.99)	0.05^f^ (0.03)	1.06 (1.00-1.12)

^a^Unstandardized parameter estimate.

^b^SE: standard error.

^c^OR: odds ratio.

^d^CI: Confidence Interval.

^e^Not available.

^f^Represents significant findings, *P*<.05.

^g^Represents marginally significant findings, *P*<.10.

### Prior Telemental Health Resource Use

Overall, 447 students reported using 1 or more of the 4 TMH tools, with most (318/447, 71.1%) of this group using only 1 type. Anonymous online chat (189/2523, 7.49%) and self-help apps or websites (191/2616, 7.30%) were the most common, followed by the crisis text line (158/2615, 6.04%) and online counselor (92/2652, 3.46%). In review of zero-order correlations involving TMH use, suicidality had the largest correlations with all 4 types (TMH website: *r*=.28, chat: *r*=.28, counselor: *r*=.27, text line: *r*=.34). Other equivalent correlations were between anxiety and self-help app or website use (*r*=.28), and between depression and online anonymous chat (*r*=.29); all other correlations were lower. As displayed in Table 4, students who had seriously considered suicide (TMH website: *P*=.003, chat: *P*=.03, counselor: *P*=.002, text line: *P*<.001) or identified their gender as “other” (TMH website: *P*=.051, chat: *P*<.001, counselor: *P*<.001, text line: *P*=.002) were more likely to report previous use of each of the 4 types. Depression predicted previous use of anonymous online chat (*P*<.001) and crisis text line (*P*=.01). Anxiety predicted previous self-help app or website use (*P*<.001); stress predicted previous use of an online counselor or therapist (*P*=.02). Female students were more likely to have used a self-help app or website than males (*P*=.02); males were more likely to use the crisis text line (*P*=.04) than females.

**Table 4:** Ordinal logistic regression analyses for combined variables predicting previous telemental health tool use (app or website, anonymous online chat, online counselor, crisis text line).

Model and predictors			B coefficient (SE^a^)	Odds ratio (95% CI^b^)
**App or website (n=2404)**				
	Intercept 1		–2.42^c^ (0.14)	—^d^
	Intercept 2		–3.02^c^ (0.15)	—
	**Gender**			
		Male versus female	–0.32^c^ (0.14)	0.73 (0.55-0.96)
		Other versus female	0.59^e^ (0.30)	1.80 (1.00-3.26)
	Suicide: Yes		0.46^c^ (0.16)	1.59 (1.17-2.16)
	Anxiety		0.25^c^ (0.05)	1.28 (1.17-1.41)
**Anonymous online chat (n=2319)**				
	Intercept 1		–2.46^c^ (0.13)	—
	Intercept 2		–3.12^c^ (0.14)	—
	**Gender**			
		Male versus female	–0.15 (0.14)	0.86 (0.66-1.13)
		Other versus female	1.06^c^ (0.29)	2.89 (1.64-5.07)
	Suicide: Yes		0.36^c^ (0.17)	1.43 (1.03-1.99)
	Depression		0.28^c^ (0.05)	1.33 (1.20-1.47)
**Online counselor (n=2119)**				
	Intercept 1		–3.83^c^ (0.31)	—
	Intercept 2		–4.38^c^ (0.32)	—
	Race: Not white		0.53^c^ (0.19)	1.70 (1.18-2.47)
	**Gender**			
		Male versus female	0.19 (0.20)	1.21 (0.81-1.80)
		Other versus female	1.49^c^ (0.39)	4.42 (2.04-9.57)
	Suicide: Yes		0.69^c^ (0.23)	2.00 (1.29-3.12)
	Stress		0.09^c^ (0.04)	1.09 (1.01-1.18)
**Crisis Text Line (n=2294)**				
	Intercept 1		–2.70^c^ (0.14)	—
	Intercept 2		–3.58^c^ (0.15)	—
	Race: Not white		0.66^c^ (0.13)	1.94 (1.51-2.50)
	**Gender**			
		Male versus female	0.27^c^ (0.13)	1.31 (1.01-1.70)
		Other versus female	0.98^c^ (0.32)	2.67 (1.43-5.00)
	Suicide: Yes		0.88^c^ (0.16)	2.41 (1.74-3.32)
	Depression		0.14^c^ (0.05)	1.15 (1.04-1.27)

^a^SE: standard error.

^b^CI: Confidence Interval.

^c^Represents significant findings, *P*<.05.

^d^Not available.

^e^Represents marginally significant findings, *P*<.10.

## Discussion

### Results and Prior Work

Youth in this high school sample reported high rates of anxiety and depression symptoms and suicidal ideation that were consistent with national averages among teenagers (31% [7], 12.8-31.5% [5,6], and 17.2% [6], respectively). In addition, more than 35% reported high stress (ie, ≥8 out of 10) in the last month. With regard to their usage of TMH, approximately 16% of youth had utilized 1 or more types of TMH resources. This number may seem low, but it is important to consider within context, as not all students have a need for these resources. Approximately half of the sample (1378/2789, 49.4%) indicated high stress, moderate or high depression or anxiety, and/or suicidal thoughts. However, as we asked about symptoms within constrained time periods but TMH usage at any time, and some students may have accessed traditional mental health care and not TMH, we did not expect these measures to be congruent. However, our prevalence statistics for stress, anxiety, and depression provide some evidence on the percentage of youth who would be likely to need such resources.

Prior research has found similarly low TMH usage rates: in 1 recent study of patients who were older than our sample (average age=57.5 years), but *all* had a documented need for mental health care, only 10% had previously used TMH apps, but over 70% were interested in doing so once informed of them [71]. This pattern of increased interest in TMH after receiving education has been noted in other research, as well [46,47]. Our sample had not necessarily received formal education on these resources before the assembly, and TMH does not encompass prototypical treatment options (ie, visiting a therapist in person or taking medication) of which they would likely be aware. We did not ask about students’ prior awareness of these resources, only their use, but in a recent longitudinal study of college students, only 10% had initially used a self-help website and even less could identify one by name; however, both use and awareness increased throughout the study period [56].

Depression and anxiety symptoms occurred at higher rates among female students, consistent with national trends [1-4,6]. However, female students were not more likely to have used anonymous chat or an online therapist than males, though they were more likely to access self-help resources, whereas males were more likely to have used the crisis text line. In addition, although the number of gender nonconforming students was small, they were more likely than those who identified as male or female to experience stress and symptoms of anxiety and depression, as well as use every form of TMH. White students reported experiencing more anxiety symptoms than minorities, and they were more likely to talk about their stress or problems with parents or friends and were less likely to talk to no one. However, minority students were more likely than white students to have used an online counselor or the crisis text line. Combined, these findings underscore the importance of measuring gender, race, and ethnicity in an inclusive way so that these relationships are more thoroughly explored and at-risk students might be better identified, educated, and treated.

In general, teens who reported issues with their mental health were more likely to have utilized TMH resources than those who did not, indicating that those who are struggling are attempting to find help and TMH has potential to serve these groups. Teens experiencing suicidality were more likely to have used all 4 categories of TMH resources, those with depressive symptoms were more likely to have used anonymous chat and the crisis text line, and those with higher stress were more likely have used an online therapist. A related theme that emerged, reflecting previous findings [8,13-15,20,21], was self-reliance and its correlation with depression and suicidality. Teens who reported depressive symptoms, higher stress, and/or suicidal ideation were less likely to report they could speak openly and honestly with their parents about problems, and more likely to have no one to talk to. Nearly 20% of students in total reported talking to no one when struggling, including 22% of prior TMH users, in line with previous findings that teens who endorse self-reliance are more likely to utilize anonymous Web-based support for problems [20]. These teens may feel more comfortable using a phone or computer to discuss mental health than, for example, asking their parent to see a therapist [15,20,43] and may benefit from the anonymity and relative independence afforded by TMH [72].

In contrast, teens reporting anxiety, as well as those who did not report any mental health symptoms, were more likely to talk to parents or friends about their problems and less likely to talk to no one. Overall, they had not used the full scope of TMH resources compared with those struggling with depression and suicidality, although they were more likely to have tried self-help apps. Together, this collection of preferences related to symptom type and available confidants represents an important consideration for researchers and developers of TMH services. Future research could further evaluate these unique preferences among symptom groups, and, assuming preferences remain, engage these target end users in participatory design and usability testing related to the specific types of apps they have shown preferences for. For example, those designing services with the goal of helping with depression and stress management could focus on evidence-based services that allow anonymous disclosure (ie, online therapy and anonymous chat) and allow these potential users to give feedback on their experiences using prototypes of the service before it is released. Services designed to reach those with anxiety could use a self-help model, and all services could potentially include information about resources (both tech-based and in local communities) to help those considering suicide.

### Future Directions

Nearly a third of the students in our study who reported recent anxiety or depression symptoms, high stress, or suicidal ideation also reported prior use of a TMH resource. However, we did not investigate in detail whether they found TMH helpful, although this is an important area of consideration for future study. In asking about prior use, we allowed students to select “yes, and it was helpful” or “yes, but it was not helpful,” which were collapsed into 1 answer of “yes” for most analyses. These responses were split nearly evenly overall (49/51%, respectively), with little variation across resource categories. However, these data alone do not provide much insight without further information regarding exact services participants were using, how they hoped to benefit from them, and why they were unsatisfied with their experience. Other research has suggested that insufficient personalization of resources is likely a factor affecting use but emphasized the difficulty of drawing these conclusions merely from survey data [71]. This reinforces the need for evidence-based and tailored resources [30], along with education about them, as discussed in the Objectives of this study.

Even when effective TMH resources are created, there should be continued work to integrate them into the health care system, likely in conjunction with face-to-face care, as this has not yet been sustainably executed on a large scale [44,57]. When attempting to integrate TMH technologies into formal health care, it is important to engage the target audience in user-centered design processes to further ensure efficacy and sustainability [45]. As stigma is a major barrier to traditional mental health treatment [8,13-17], the relative flexibility, immediacy, and anonymity of TMH may mitigate this concern [13]. Prior research also suggests that successful Web-based self-disclosure, in general, may lead to self-disclosure in person [73]; thus, future research could additionally explore TMH as a bridge between self-reliance and professional help. In addition, many prior studies have revealed insufficient knowledge about both traditional mental health care and TMH [13,17,18]. To effectively locate and utilize TMH resources, youth must be able to identify their symptoms and find reputable avenues of support. Although the events connected to this study educated participants about available sources of tech-based help, barriers to TMH use (including lack of awareness, stigma, and feelings of trust) should be a focus of future research.

TMH also has the potential to play an important role in suicide prevention—youth in this study who had considered suicide were more likely to have used all categories of resources. Given the disparity between urban and rural suicide rates [74], and the fact that individuals in rural locations may particularly benefit from TMH resources [4,17,43] because of geographic isolation from traditional treatment, TMH may be a useful addition to suicide treatment and prevention efforts, which, in light of current trends, need revision or supplementation [8,17]. Our sample included only suburban and rural schools, and we did not evaluate the urban or rural status of individual students; thus, we are unable to elucidate the usefulness of TMH among these different groups. However, we believe it is an important direction for future research.

Finally, participants in prior research have cited data protection, information security, and anonymity as important concerns related to health care delivered via apps or websites [30]; therefore, TMH resources must be able to guarantee that personal health information will be safe [43,45]. With regard to public buy-in, concerns related to teen social media and internet use persist, as anonymous online behavior may increase susceptibility to cyberbullying and leave individuals more vulnerable in a suicide attempt, without a real-life support network to intervene [20]. However, extant literature suggests that benefits of TMH outweigh concerns [38], many of which could be mitigated by appropriate design, moderation, or professional involvement [43,45,50,]. Future research should continue to consider these safety-related factors when developing new interventions.

### Limitations

Our sample included only students enrolled in and present at school, whose parents had not opted out. Several variables (ie, male or other gender, minority race, higher depression level) related positively to nonresponse, potentially limiting data for topics particularly sensitive to these individuals. The setting of the survey (ie, an auditorium or gymnasium in which students were seated near each other and could not be truly prevented from speaking) could have contributed to nonresponse or skewed responses toward lower reporting of stigmatized topics. Conversely, the group setting may have kept other students engaged. In addition, the themed presentation may have influenced students to respond either positively or negatively to the TMH-related questions, if they assumed we were seeking positive response and chose to fulfill or deny this expectation. However, we do not believe these limitations would necessarily create strong trends in any specific direction, nor would they be entirely mitigated in a different setting (ie, classroom or computer lab).

In addition, the number of students reporting their gender as “other” was small, but gender-nonconformity was associated with higher rates of depression and anxiety symptoms, suicidal ideation, stress, inability to discuss stress or problems openly and honestly with a parent (or anyone), and prior use of all 4 TMH categories. We did not record sexual orientation, but given the consistency of gender nonconformity as a predictor, future research should explore both gender and sexual minority status in relation to mental health help-seeking. These 2 groups, which sometimes overlap, face similar sets of social and familial challenges and may uniquely benefit from the lack of required parental involvement involved in TMH help-seeking.

This high school survey was part of a larger study about TMH usage that also included college students; thus, the PHQ-4 was used for consistency among both groups, although it has not been specifically validated with adolescents. However, over 37% of our sample for this study was aged 17 to 19 years (for whom the PHQ-4 has been validated [65]), and the 2 measures from which the PHQ-4 was created (the PHQ-2 and GAD-7) have been validated among adolescents aged 13 to 17 years [66,67]. Given these factors, along with the expected rates of depression and anxiety reported by our sample, we believe the PHQ-4 was an acceptable choice for the 13 to 19 years age group despite lacking validation in its exact 4-item format for the younger students.

### Conclusions

Overall, our results indicate that teenagers experiencing mental distress are utilizing existing TMH resources at a moderate rate consistent with extant literature. Type of resource usage correlated with mental health and demographic variables, providing a framework for future research and targeted resource development. Suicidality and gender nonconformity predicted use of all 4 categories of resources; depression, anxiety, and stress all predicted use of at least 1 unique type. In addition, suicidality, depression, and stress were correlated with lacking confidants with whom to discuss stress or problems, whereas those with anxiety were less likely to report this. As the mental health field progresses toward electronically-based care, it is important to consider findings such as these to provide appropriate interventions that target specific populations for effective and tailored care or supplementation of care [60]. Particularly, access to evidence-based resources that use varying methods of engagement based on symptoms or preference [13,29] and/or allow students to discuss their issues informally, on their own time, mutually, and anonymously [17,21,28,43,46,49,50] may be promising routes for providing support for specific symptoms, stressors, or demographics among youth who are in need of mental health interventions.

## Abbreviations

PHQ: Patient Health Questionnaire
TMH: telemental health
